# The clinical utility of gene expression examination in rheumatology

**DOI:** 10.31138/mjr.28.3.116

**Published:** 2017-09-29

**Authors:** Elena Tchetina, Galina Markova

**Affiliations:** Immunology and Molecular Biology Laboratory, Nasonova Research Institute of Rheumatology, Moscow, Russia

**Keywords:** rheumatoid arthritis, gene expression, TNFα, type I IFN-response genes, response to therapy, proteases, pain molecular markers

## Abstract

Rheumatoid arthritis (RA) is a chronic inflammatory disease with unknown etiology that affects various pathways within the immune system, involves many other tissues and is associated with pain and joint destruction. Current treatments fail to address pathophysiological and biochemical mechanisms involved in joint degeneration and the induction of pain. Moreover, RA patients are extremely heterogeneous and require specific treatments, the choice of which is complicated by the fact that not all patients equally respond to therapy. Gene expression analysis offer tools for patient management and personalization of patient’s care to meet individual needs in controlling inflammation and pain and delaying joint destruction.

## INTRODUCTION

Rheumatic diseases (RDs) are a group of musculoskeletal disorders characterized by inflammation, swelling and pain in joints and muscles, and other systemic features caused by immune-mediated attacks on self-antigens.^1^ More than 100 disorders are included in the National Data Bank for Rheumatic diseases.^2^ Because both genetic and environmental factors are involved,^3^ the precise cellular and molecular mechanisms leading to rheumatic disease development and organ damage are unclear at present.

Rheumatoid arthritis (RA) is a prototypic chronic inflammatory rheumatic disease characterized by synovial hyperplasia, pannus formation, mononuclear cell infiltration, articular cartilage and bone erosion, and joint destruction. Synovial tissue dysfunction in RA worsens lubrication and nutrition for articular cartilage.^4^ Synovial tissue is invaded by macrophages, fibroblasts, and activated lymphocytes. T-lymphocytes are involved in the production of a wide range of proinflammatory cytokines, predominantly in the tumour necrosis factor (TNF) and interleukin (IL) superfamilies, as well as growth factors.^5^ B-lymphocytes are associated with the production of autoantibodies, such as rheumatoid factor (RF) and anti-cyclic citrullinated peptide antibodies (ACPA).^6^ RA is a heterogeneous condition. Heterogeneity is ensured by various factors, including ACPA and/or RF positivity,^7^ the pace of the disease course,^8^ and variability in response to treatment.^9^ This might suggest the involvement of different pathophysiological mechanisms. Therefore, adequate treatment of RA requires specific diagnostic tests and optimal biomarkers to distinguish between different manifestations of the disease. However, treatment of RA is mainly focused on relieving symptoms, because no curative therapy is available; complications in multiple organs limit the long-term administration of therapeutic agents in RA patients.^10^ Therefore, RA treatment relies on the modulation of autoimmune abnormalities and downstream inflammatory cascades, primarily the TNFα pathway.^11^

Nearly every aspect of the RA disease phenotype can be described in the pattern of genes and proteins expressed in the patient.^1^ The human genome contains approximately 20,000 protein-coding genes, encoding complex signaling pathways that are activated under inflammatory conditions and produce alterations in cellular metabolism affecting growth and survival.^12^ The fundamental rationale for gene expression assessment is that perturbations in a biological system caused by a disease lead to immediate alterations in gene expression. Therefore, analysis of the gene expression changes that are associated with the disease might permit identification of specific metabolic pathways related to its pathogenesis. Alternatively, specific patterns of gene expression might mirror the response of a biological system to disease status or therapeutic intervention; as gene expression is affected not only by genetics, but also by lifestyle factors including diet, drugs, exercise, gut microbiota, health-to-disease status, hormonal homeostasis and age.

Although analysis of gene expression in tissue samples from the affected organs reveals genes that are primarily involved in the disease, this approach is not suitable for large cohorts of patients. Moreover, owing to the systemic nature of many RDs and communication between the systemic and organ-specific compartments, whole blood and peripheral blood mononuclear cells (PBMCs) analysis could be more appropriate especially for identification of biomarkers for personalized therapy.^1^

In contrast to the researcher’s interest in genes, proteins, cell signaling, metabolic pathways, and structural aspects, the patient is focused on pain, functional limitations, aesthetic damage due to bony proliferations and loss of daily and social activities.^13^ In view of this, patients are mostly interested in the prognosis of the efficacy of treating inflammation, pain control, and blockade of their joint destruction. Therefore, as gene expression is the earliest marker of body changes in response to the disease or treatment, examination of the genes controlling these matters at baseline and over the course of the disease might be a helpful prognostic instrument in the clinical setting.

## PROGNOSTIC VALUE OF EXAMINING BASELINE PROINFLAMMATORY CYTOKINE GENE EXPRESSION

As several drugs have been developed to control RA disease activity, the clinical rheumatologist is required to choose a therapeutic approach that will produce the best treatment result based on the patient’s disease status. The complexity of manifestation patterns in RA and the variety of the genetic contributors and mediators in each patient complicates a predictive test. However, as inflammation is a hallmark of RA, monitoring proinflammatory cytokine gene expression might be a reasonable approach.

TNFα and type I interferons (IFNs) are considered to be common denominators of RDs.^1^ TNFα expression is increased in various immune-mediated diseases such as RA, ankylosing spondylitis (AS), osteoarthritis (OA), and psoriatic arthritis (PsA)^14^ while IFN-response genes upregulation was observed in patients with ACPA-negative RA, systemic lupus erythematosus (SLE), myositis, and scleroderma (SSc).^15^ Indeed, type I IFNs are early mediators of the innate immune response that affects the adaptive immune response by direct and indirect actions on dendritic cells, T cells, B cells, and natural killer cells; they could affect the initiation or amplification of autoimmunity and tissue damage.^1^ Moreover, the upregulation of proinflammatory cytokine gene expression is expected, as RA is considered a TNFα-driven disease.^16^ TNFα-blocking agents are often efficient in ameliorating RA manifestations, particularly in ACPA-positive subjects. In addition, *in vitro* studies have demonstrated that TNFα can down-regulate the effects of type I IFNs and vice versa.^17^

### Tumour Necrosis Factor

Several studies reported that RA patients with higher levels of synovial inflammation and synovial TNFα expression^18^ and increased expression of inflammation-related IL2 receptor-beta, SH2 domain 2A, and GOS2 in peripheral blood respond better to anti-TNF blockade.^19^ Moreover, high baseline gene expression of TNFα in patients whose serum C-reactive protein (CRP) decreased to the levels observed in normal subjects after treatment appears to be a useful marker for infliximab treatment efficacy.^20^ The negative correlations between baseline TNFα gene expression and the number of tender and swollen joints measured at the end of methotrexate (MTX) treatment in early RA patients suggests a predictive potential of TNFα gene expression for prognosis of the efficacy other anti-rheumatic drugs.^21^

The observation that increased expression of proinflammatory genes in responders normalized faster than in non-responders over the course of anti-TNF treatment^22^ might be associated with the downregulation of various immune-related pathways, including inflammation.^23^ Therefore, a high baseline level of TNFα gene expression might help identify antirheumatic therapy responders.

### Type I Interferons

Gene expression levels of proinflammatory cytokines can vary among RA patients. For example, upregulation of type I IFN-response genes was observed in peripheral blood in about a half of RA patients (IFN “high” patients), although no clinical differentiation between IFN “low” and “high” patients was noted.^24^ The IFN signature was observed equally often in seropositive and seronegative RA patients with equal plasma levels of TNFα. Therefore, the presence of TNFα and IFNs are not mutually exclusive, but might indicate the simultaneous involvement of multiple immune mechanisms in RA.^9^

The IFN “high” group exhibited significantly upregulated pathways involved in coagulation and complement cascades, and fatty acid metabolism compared to healthy controls. At the same time, a “high” type I IFN signature was associated with a lower level of disease activity and the persistence of ACPA after TNF blockade.^25^ In IFN “low” patients, the expression of type I IFNs was equal to that of control subjects.

The level of type I IFN bioactivity affects the clinical response to TNFα blockade in RA patients, although these results have not always been consistent.^26^ A better clinical response to TNF-antagonists associated with high baseline plasma levels of type I IFN^25^ might result from increased TNFα expression and an overall higher level of inflammatory activity in patients with “high” IFN signatures compared to “low” IFN signature subjects. In addition, the anti-inflammatory effects of high levels of IFNβ might be involved, as a higher IFNβ/IFNα ratio prior to initiation of TNF blockade result in a better clinical response.^27^

At the same time, patients with a “low” baseline IFN signature who did not respond to anti-TNF blockade, showed an increase in type I IFN response gene expression by the end of treatment,^28^ indicating that neutralization of TNFα in these patients favours the upregulation of genes that were previously silenced by TNFα.^29^ Alternatively, the upregulation in IFN bioactivity has also been suggested to be deleterious in RA or to represent a failed attempt to counter-regulate inflammation.^25^

Anti-IL6 treatment can decrease the expression of numerous chemokine and T cell activation genes in RA synovium.^30^ IL6-blocking therapy in RA is efficient when type I IFN response gene expression in the PBMCs was increased.31 However, this observation contradicts previous reports that type I IFNs can enhance IL-6 signaling by providing docking sites for STAT1 and STAT3 on the phosphorylated IFNα receptor 1 (IFNAR1) in close proximity to the gp130 chain of the IL-6 receptor.^32^ As both anti-IL6 and anti-TNFα, blocking therapies appear to be more efficient when IFN activity is increased, the molecular and cellular mechanisms underlying the therapeutic effects of IL6 and TNFα antagonists may share a similar pathway in the pathophysiology of RA.^33^

A good response to anti-B cell treatment by rituximab (RTX) was observed when genes involved in inflammation, primarily nuclear factor kappa B (NFkB) - and transforming growth factor (TGF) β-signaling were upregulated and IFN-response genes were downregulated at baseline.^9,34^ Deleterious effects of type I IFNs are associated with the enhancement of B-cell survival through direct stimulation of B-cells or production of B-lymphocyte stimulator (BLyS) and a proliferation-inducing ligand (APRIL)^35^ and by stimulation of T-cells and dendritic cells.^36^ At the same time, IFNβ can reduce secretion of proinflammatory cytokines, such as IL-6, matrix metalloproteinases (MMPs), and prostaglandin E_2_ by fibroblast-like synoviocytes. It possesses anti-angiogenic properties and can inhibit osteoclastogenesis. Hence, IFN signature activation in RA synovium could be a reactive attack to limit inflammation.^37^

In addition, dissimilar gene clusters and distinct molecular signatures specifically expressed during early or long-standing RA suggest the involvement of different pathophysiological mechanisms in the disease course as a function of disease progression.^38^ In view of this, studies of early RA patient synovial tissue showed higher levels of TNFα-related gene expression, while long-standing RA patients had higher levels of IFN-response gene expression correlated with the downregulation of the metalloproteinase inhibitor gene and total protein biosynthesis.^39^

Therefore, high TNFα and/or type I IFN-related gene expressions in peripheral blood and/or synovium at baseline might suggest a better response to anti-TNFα or anti-IL6 treatments in RA patients, while low expression of type I IFN-response genes suggests a better response to anti-B cell therapy in RA patients.

The disturbance in type I IFN signaling both in peripheral blood and target organs has been demonstrated in other rheumatic diseases, such as SLE, where IFNα plays a primary pathogenic role in autoimmunity and disease pathogenesis.^40^ For example, rontalizumab, an anti-IFNα antibody, produced significant improvements in disease activity and flare manifestations in SLE subjects with a “low” IFN-signature gene expression compared to “high” IFN patients.^41^ Disturbances in type I IFN expression observed in the blood of patients with Sjögren’s syndrome,^42^ fibromyalgia,^43^ psoriatic arthritis,^44^ and OA^45^ also suggest a potential prognostic importance of the expression of genes in clinical practice in the above conditions.

## ASSOCIATION OF JOINT DESTRUCTION WITH UPREGULATION OF PROTEASES

Radiographic progression of bone destruction is associated with a poor prognosis in RA disease and is related to increased bone resorption and fracture rates.^46^ Bone erosions have been correlated with disease severity and with long-term disability in RA patients.^47^ Moreover, the Sharp/van der Heide erosion score is considered the strongest potential predictor for biologic agent dose reduction or discontinuation.^48^ Therefore, prevention of bone erosions is an important therapeutic endpoint in RA treatment.

Damage to the bone in RA is defined by joint erosion, while cartilage injury is approximated by measuring joint space narrowing.^49^ Animal studies have shown a close relationship between cartilage loss and erosion of subchondral bone in RA.^50^ Although bone erosions are considered critical indicators of disability in RA patients, recent studies suggest that articular cartilage destruction occurs early in the disease and may be more important in the assessment of irreversible physical disability.^51^ At the same time, accumulated evidence suggests that bone marrow lesions occur at an early stage of RA and may precede synovitis.^52^ Moreover, early joint destruction is more rapidly progressive than at the later RA stages.^53^ The extent of joint damage progression in RA is primarily related to the degree of the inflammatory process as revealed by joint swelling, the acute phase response, and the level of the disease activity.^54^ Chronic inflammation in RA leads to focal bone erosions within inflamed joints and to generalized osteoporosis in the axial and appendicular skeleton.^55^ In addition, chronic inflammation is often associated with the generation of specific immune responses and with concurrent tissue damage and repair as opposed to the sequential progress from insult to resolution observed in acute inflammation.^56^ However, damage might increase despite the absence of clinical activity (silent progression),^57^ owing to a low sensitivity of clinical joint assessment, subclinical synovitis or systemic effects caused by activity in other joints.^58^ On the other hand, the ability of bone erosions to repair in RA is limited because of the persistence of residual synovial inflammation despite of clinical remission.^59^ Even when osteoclast activity is inhibited by therapeutic interventions, only approximately 10% of RA patents repair erosions.^60^

Bone remodelling requires balanced activity between bone-resorbing osteoclasts and bone-forming osteoblasts. Receptor activator of nuclear factor kappa-B ligand (RANKL) is an essential factor for osteoclast differentiation while upregulation of Runt-related transcription factor (RUNX2) in osteoblast precursor cells is required for osteoblast formation.^61^ Synovial inflammation inhibits local osteoblast differentiation, resulting in the presence of immature osteoblasts at the sites of inflammation. This is evidenced by a lack of expression of osteoblast maturation markers, alkaline phosphatase, and osteocalcin.^62^ In contrast, inhibition of inflammation favours osteoblast maturation and bone erosion repair.^63^ RANKL expression in synovial B cells rather than T cells is responsible for formation of osteoclasts and erosions during collagen-induced arthritis in animals^64^ and structural damage of inflamed joints in humans.^65^ Inflammation promotes osteoclast differentiation and bone destruction.^66^ This is supported by the observation of lower rates of bone formation at bone surfaces adjacent to inflammation site compared to bone surfaces adjacent to normal marrow.^62^ TNF engages TNF-receptor type I on the surface of osteoclast precursors, stimulating their differentiation into osteoclasts.^67^ Therefore, it is not surprising that TNF inhibition can halt radiographic joint destruction despite remaining active disease.^68^ In contrast, some studies have shown that alternative osteoclastogenic RANKL-independent pathways might be functional in autoimmune arthritis^69^ as specific inhibition of osteoclast activation that does not address joint inflammation reduced joint destruction in RA.^70^

However, all these activities eventually merge at proteinase level; proteinases are the endpoint agents involved in bone and cartilage matrix destruction as they are responsible for enzymatic cleavage of peptide bonds.^71^ They are also involved in processing precursors related to the synthesis of collagen, immune functions, development, and apoptosis, as well as in catabolic reactions during healthy tissue remodelling and their altered activity is associated with cartilage destruction and bone erosion in RA.^72^ Therefore, proteinase activity must be carefully controlled to avoid inappropriate degradation of proteins. The most important proteinases involved in bone destruction are cathepsins B, L, and K, which are upregulated in synovial fibroblasts in RA patients.^73^ Cathepsin K, a cysteine proteinase, is crucial in bone remodelling and is predominantly expressed in osteoclasts as well as in fibroblasts and macrophages in RA joints.^74^ Recent animal studies have shown that genetic deletion of cathepsin K causes significant reduction in inflammation and bone erosion within RA joints.^75^

The MMPs are major mediators of cartilage destruction. The MMP subfamily of metalloproteinases are capable of cleaving extracellular matrix (ECM) components and other active molecules.^76^ For example, collagenase MMP-1 is responsible for degradation of collagen types I, II, and X.^77^ MMP-3 can degrade various components of the ECM including aggrecan and collagen types II, IV, IX, and XI, and has the potential to activate MMP-1 and pro-MMP9. Increased production of different MMPs was observed in the synovial fluid and synovial fibroblasts in inflamed joints.^78^ Macrophage migration inhibitory factor is mainly produced by macrophages in response to various inflammatory stimuli and has been shown to upregulate expression of MMP-1 and -3 in cultured synovial fibroblasts from RA patients,^79^ while MMP-9 and MMP-13 expression in RA joint fluid is significantly associated with VEGF and might be involved in angiogenesis.^80^ Proteinases are also involved in joint destruction in OA,^81^ spondyloarthropathies,^82^ SSc,^83^ and SLE.^84^

Levels of MMP-1 and MMP-3 in the serum of RA patients are correlated with disease activity.^85^ Alternatively, successful treatment of RA with leflunomide, MTX or anti-TNFα antibodies is associated with downregulation of MMPs in serum.^86^ In addition, MMP concentrations might predict functional and radiographic progression, as in early untreated RA patients baseline serum levels of MMP-1 and MMP-3 correlated with erosive disease during the first 12 months.^87^

Blood-based gene expression, being the earliest response to alterations in the cellular environment, might also contribute to joint destruction assessment; a correlation between gene expression at diseased sites and in the peripheral blood was reported for matched subjects.^88^ Moreover, the increase in erosion numbers after MTX treatment in seropositive early RA patients was accompanied by upregulation of MMP-9 and cathepsin K gene expression in the blood.^21^ In contrast, the treatment of seropositive RA patients with RTX, which did not augment erosion numbers or joint space narrowing indices, was associated with decreased MMP-9 and cathepsin K gene expressions in the blood.^89^

In addition, blood-based gene expression examination showed that radiographic severity monitored by erosion assessment in RA patients was associated with upregulation of IFN- and TGFβ-signaling and apoptosis activity, and with downregulation of oxidative phosphorylation and mitochondrial function both at baseline and after three years of the disease.^90^ Another study identified a set of 14 genes including proinflammatory and growth arrest-related genes that predict severity of the disease that were upregulated in peripheral blood.^91^

## MOLECULAR APPROACHES FOR RHEUMATIC PAIN MANAGEMENT

Pain is the most dominant and disabling symptom reported by RA patents at every stage of the disease. Therefore, pain relief is expected from every disease treatment for most patients.^92^ Treatment of pain in RA includes nonpharmacologic methods such as self-management programmes and exercise^93^ and pharmacologic therapies using medications for treatment of chronic pain syndromes.^94^ However, pharmacological choices for RA pain management are limited and inadequate.^95^ At the same time, reduction of pain by 30% is considered as a good treatment result although it is associated with maintenance of a significant number of symptoms in approximately 60% of RA patients.^96^

### Pain mechanisms

Pain is classified as an acute or chronic in RA. Acute pain is primarily nociceptive and is linked to acute inflammation. It is intermittent and sharp, localized at the site of injury and resolves with the resolution of inflammation. It is associated with a response to inflammatory molecules in the first-order somatosensory neurons, which transmit a signal to the brain via the dorsal horn of the spinal cord.^97^ Chronic pain represents a combination of pain arising from tissue destruction and is sustained by activation of neuropathic pain mechanisms involving nerve damage or dysfunction.^98^ Chronic pain might result from acute pain that persists due to inadequate repair processes, producing functional tissue that might differ in its cellular or matrix composition from that which preceded the insult.^56^ Another type of chronic pain involves the disconnection of the pain-generating process from the initial tissue injury (neuropathic pain).^99^ Mechanistically, neuropathic pain involves a peripheral and central sensitization,^100^ where sensitization represents a process by which repeated administration of a stimulus results in the progressive amplification of a response.^101^ Peripheral sensitization is associated with reduction of a threshold and augmentation of responsiveness of nociceptors.^102^ In contrast, central sensitization includes altered brain processing of sensory inputs, descending anti-nociceptive dysfunction, increased activity of pain facilitatory pathways, temporal summation (wind-up) and long-term potentiation of neuronal synapses in the anterior cingulate cortex.^103^ The mechanisms of chronic pain development are not completely clear at present and might involve a disturbance in apoptotic cell death pathways, pathological reduction of supraspinal inhibitory activity^104^ or increased communication between small satellite glial cells (SGCs) and between neurons and SGCs after peripheral noxious stimulation.^105^

Sensory studies have shown that RA patients demonstrate amplified responses to pain,^106^ which is commonly reported and the most impairing stressor in RA.^107^ Elevations of daily stress among RA patients are associated with increases in musculoskeletal tenderness, IL-6 levels and disease activity.^108^ Forty-seven percent^109^ of RA patients exhibit lower pressure pain thresholds and enhanced sensitivity to noxious stimuli both in inflamed joints and non-inflamed tissues compared to healthy controls^110^ while widespread pain and pain hypersensitivity in 10–20% of patients is associated with poorer treatment outcomes.^111^ Moreover, a relative hyporesponsiveness of the autonomic nervous system, hypothalamic-pituitary-adrenal system,^112^ and a reduction of descending analgesic pathways in RA patients compared to healthy subjects were also noted.^113^

RA patients may sense pain before inflammation while pain may persist despite control of inflammation,^114^ as up to 60% of patients with RA continue to report pain as a major concern following adequately suppressed inflammation.^115^ This might suggest that synovial inflammation may prompt central sensitization^116^ and is consistent with two mechanisms of RA pain: a peripheral mechanism associated with inflammation and a central mechanism associated with a set of symptoms involving sleep problems, fatigue, and changes in mood.^117^ Similar pain mechanisms were observed in other rheumatic diseases, such as SLE, AS, OA, and inflammatory bowel disease.^118,119^

### Pain molecular markers identified in RA patients

Our knowledge of pain molecular markers expressed in RA patients is limited. These are several molecular markers of peripheral nociception that are often seen in the synovial fluid of RA patients, including acid-sensing ion channels (ASICs), which are activated by decreased extracellular pH.^120^ Transient receptor potential cation vanilloid (TRPV1) channels are implicated in arthritis as they were found both on primary afferent nerves in the periphery^121^ and on synoviocytes.^122^ In addition, osteopontin levels in patient synovial fluid positively correlated with the severity of joint pain after ACL rupture.^123^ Nerve growth factor (NGF) expression and sensory nerve growth might link osteochondral angiogenesis to pain in RA^124^ as increased levels of NGF have been reported in RA synovial fluid.^125^ Moreover, lymphotoxin-beta receptor gene expression in RA patient synovium is positively correlated with Pain Visual Analogue Scale (VAS) scores.^126^ At the same time, concentrations of lipids capable of decreasing sensory neuron excitability, such as endocannabinoids and lipid agonists of peroxisome proliferator-activated receptor (PPAR)α^127^ are reduced in RA patient synovial fluid compared to healthy controls.^128^ However, with prolonged and enhanced inflammation, the immune and peripheral nervous system could upregulate expression of opioid receptors and their ligands in sensory nerves to counterbalance pain and inflammation. For example, upregulated expression of beta-endorphin, met-enkephalin and opioid receptors was noted within synovial sub-lining cells, macrophages, lymphocytes, and plasma cells in RA patients.^129^

Central pain processing is also increased in RA patients and involves alterations in neuronal adaptive response and upregulation of thalamus, secondary sensory cortex, and limbic system activities.^130^ The involvement of central mechanisms in RA patients is evidenced by fractalkine (FKN) signalling via the CX3CR1 receptor, which mediates neuroglial communication in chronic pain states and is implicated in the development of neuropathic pain in RA.^131^ In addition, elevated expression of cytokines such as TNFα observed in the brain in RA patients^132^ may damage the CNS by favouring accumulation of extra-cellular glutamate.^133^ Alternatively, neutralization of TNFα can inhibit chronic pain in RA patients much faster than it improves the signs of inflammation such as reduction of swelling, probably by decreasing TNFα-mediated nociceptive neurotransmission (synaptic plasticity) in the spinal cord dorsal horn prior to the improvement of inflammation.^134^ Concentrations of IL-1β are also elevated in the cerebrospinal fluid of RA patients.^135^ Increased levels of IL-1 and IL-6 in the brain were previously associated with fatigue, which is positively correlated with pain in RA.^130^ In addition, IL6 upregulation was suggested to be responsible for post-surgery pain in RA patients.^136^

### Assessment of pain remaining after treatment

Discordance between inflammation and pain and the involvement of central mechanisms in generating and maintaining chronic pain in RA patients necessitates the careful development of prognostic tools for evaluating remaining pain before treatment onset in a clinical setting. Lingering pain despite a good clinical response was strongly associated with functional impairment and disability at baseline,^116^ fibromyalgia,^137^ high baseline pain, and low inflammation as evidenced by lower baseline levels of erythrocyte sedimentation rate (ESR) and C-reactive protein (CRP).^138^ Moreover, a pain DETECT questionnaire score ≥ 19, indicating central sensitization, predicts poorer treatment outcome estimated by clinical status (DAS28-CRP).^139^

A gene expression approach might permit precise identification of the molecular cause of pain in individual RA patents. For example, high residual expression of MMP-9 and cyclooxygenase (COX-2) in RA patients treated with RTX^89^ could be associated with joint pain maintenance because these gene products are associated with neuropathic pain in animal studies.^140^ In addition, pain control might be exerted by autophagy mechanisms as its induction by rapamycin, pentobarbital or morphine was accompanied by long-lasting analgesic effects, while its inhibition by chloroquine or miR-195 aggravated neuropathic pain following peripheral nerve injury in animal studies.^141^ The negative correlation between baseline expression of autophagy-related ULK1 gene and the number of tender joints at the end of therapy observed in RA patients treated with RTX^89^ was accompanied by a positive correlation between baseline ULK1 expression and the same gene expression after RTX treatment. This indicates an improved capacity of RA patients with high baseline ULK1 gene expression to maintain sufficient levels of autophagy activity for pain regulation and is supported by the observation that stimulation of autophagy was associated with suppression of clinical arthritis and inflammatory cytokine production in RA.^142^

## CONCLUSION

Gene expression examination provides new insights into the complexity of pathogenesis of rheumatic diseases and offers a basis for identification of biomarkers for future clinical applications. The studies described above indicate that several gene expression biomarkers related to major patient concerns have been already identified, including inflammation, joint destruction, and pain. Gene expression analysis is particularly useful in the clinical setting as it permits patient stratification, enabling prescription of specific drugs that could modulate differential transcriptional pathways and meet personal treatment requirements. Therefore, analysis of gene expression over the course of the rheumatic disease could potentially improve outcomes and decrease the proportion of refractory patients. Gene expression analysis could also ensure the safe use of an increasing variety of innovative medicines with different modes of action. Further independent validation in large well-powered cohorts is essential to explore future clinical implications of the gene expression approach. For this purpose, standardized procedures for sample processing, technology, data analysis and algorithms are required.

## ABBREVIATIONS

ACPA: anti-cyclic citrullinated peptide antibodies
APRIL: proliferation-inducing ligand
AS: ankylosing spondylitis
ASICs: acid-sensing ion channels
BLyS: B-lymphocyte stimulator
CRP: C-reactive protein
ECM: extracellular matrix
ESR: erythrocyte sedimentation rate
FKN: fractalkine
IFN: interferon
IL: interleukin
MMP: matrix metalloproteinase
MTX: methotrexate
NFkB: nuclear factor kappa B
NGF: nerve growth factor
OA: osteoarthritis
PBMCs: peripheral blood mononuclear cells
PPAR: peroxisome proliferator-activated receptor
PsA: psoriatic arthritis
RA: rheumatoid arthritis
RANKL: receptor activator of nuclear factor kappa-B ligand
RDs: rheumatic diseases
RF: rheumatoid factor
RTX: rituximab
RUNX: Runt-related transcription factor
SGCs: satellite glial cells
SLE: systemic lupus erythematosus
SSc: scleroderma
TGF: transforming growth factor
TNF: tumour necrosis factor
TRPV: transient receptor potential cation vanilloid
VAS: Visual Analogue Scale
